# A Comprehensive Evaluation of Alignment Algorithms in the Context of RNA-Seq

**DOI:** 10.1371/journal.pone.0052403

**Published:** 2012-12-26

**Authors:** Robert Lindner, Caroline C. Friedel

**Affiliations:** 1 Institute of Pharmacy and Molecular Biotechnology, Heidelberg University, Heidelberg, Germany; 2 Institute for Informatics, Ludwig-Maximilians-Universität München, Munich, Germany; Johns Hopkins University, United States of America

## Abstract

Transcriptome sequencing (RNA-Seq) overcomes limitations of previously used RNA quantification methods and provides one experimental framework for both high-throughput characterization and quantification of transcripts at the nucleotide level. The first step and a major challenge in the analysis of such experiments is the mapping of sequencing reads to a transcriptomic origin including the identification of splicing events. In recent years, a large number of such mapping algorithms have been developed, all of which have in common that they require algorithms for aligning a vast number of reads to genomic or transcriptomic sequences. Although the FM-index based aligner Bowtie has become a de facto standard within mapping pipelines, a much larger number of possible alignment algorithms have been developed also including other variants of FM-index based aligners. Accordingly, developers and users of RNA-seq mapping pipelines have the choice among a large number of available alignment algorithms. To provide guidance in the choice of alignment algorithms for these purposes, we evaluated the performance of 14 widely used alignment programs from three different algorithmic classes: algorithms using either hashing of the reference transcriptome, hashing of reads, or a compressed FM-index representation of the genome. Here, special emphasis was placed on both precision and recall and the performance for different read lengths and numbers of mismatches and indels in a read. Our results clearly showed the significant reduction in memory footprint and runtime provided by FM-index based aligners at a precision and recall comparable to the best hash table based aligners. Furthermore, the recently developed Bowtie 2 alignment algorithm shows a remarkable tolerance to both sequencing errors and indels, thus, essentially making hash-based aligners obsolete.

## Introduction

Novel possibilities and challenges have been introduced to genome analysis by the emergence and rapid distribution of next generation sequencing (NGS) technologies. In contrast to traditional Sanger sequencing, the NGS counterpart relies on multiple-fold coverage of each sequenced base by many short sequencing reads. What took the International Human Genome Consortium over a decade and an estimated $300 million to complete, is now feasible within one day’s time and at a fraction of this price. Due to falling sequencing costs, NGS technologies have been extended to many more applications apart from genome sequencing, in particular transcriptome sequencing and quantification.

Previously used hybridization-based methods for quantification and characterization of transcripts require careful design of the array platform and knowledge about the transcriptome under investigation. Furthermore, they suffer from cross-hybridization effects and have a limited dynamic range [1]. Earlier sequencing-based approaches to transcript quantification such as Serial Analysis of Gene Expression (SAGE) or Cap Analysis of Gene Expression (CAGE) had the advantage of providing count-based measures of transcript abundance, however due to high per-base sequencing costs, high throughput could only be achieved at the expense of small, often ambiguously mapping tag sizes. Furthermore, since transcripts were merely identified by their 3′- or 5′-terminal tags, these methods were oblivious to variations within the transcript. High-throughput RNA sequencing (RNA-seq) overcomes these limitations and provides a single methodology to assess transcript sequence, structure and abundance.

The mapping of sequencing reads to their sequence origin is the first step upon which any subsequent analyses are based. A specific challenge for the sequencing of eukaryotic transcriptomes is the mapping of reads from spliced transcripts to the genome. As read lengths increase, the number of reads spanning exon junctions increases such that alignment to an unspliced reference becomes impractical. Accordingly, a large number of mapping approaches have been developed in recent years to address this problem, including TopHat [2], SpliceMap [3], MapSplice [4], RUM [5], RNASEQR [6] and ContextMap [7]. While these approaches differ in their strategies for mapping reads crossing splice junctions or the use of only an alignment to the genome (e.g. TopHat, MapSplice and ContextMap) or also to the transcriptome (e.g. RUM and RNASEQR), all of these require specialized alignment algorithms to actually align the sequencing reads to the genome or transcriptome. For instance, RUM [5] and RNASEQR [6] start with read alignments to the reference transcriptome and genome and then identify novel splice junctions only from the reads not aligned in the first step.

Due to this importance of read alignment for any application of NGS technologies, many software tools for short read alignment have been published. Although standards such as MAQ [8] and in particular Bowtie [9] exist in the field, they are not unchallenged and many more alignment algorithms have been developed (see the Methods section for an overview). Remarkably, however, developers of mapping algorithms for RNA-seq have mostly ignored more recently published alignment algorithms and almost exclusively use Bowtie as internal alignment program. While the usage of the same alignment algorithm in all of the mapping approaches listed above makes it easier to perform an unbiased comparison of the different strategies in identifying spliced reads (see [5] and [7] for recent evaluations of mapping algorithms), it completely overlooks the possibility of improving both alignment accuracy and runtime performance of any of these strategies by exchanging the internal aligner. This highlights the need for a comprehensive comparison of alignment algorithms with a particular focus on RNA-seq data.

Although newly proposed alignment algorithms are generally compared against a few selected other algorithms, there exist few comparative studies on a wider range of algorithms which could provide some guidance in the choice of the algorithm. Recently, Ruffalo *et al.* published a study comparing accuracy and runtime on genome alignments for increasing genome sizes and eight commonly used alignment algorithms [10]. Unfortunately, only algorithms based on reference indexing either by hash tables or FM-index were included and no algorithms using read indexing were evaluated. Furthermore, accuracy was only evaluated in terms of correctly and incorrectly mapped reads and the sensitivity of the algorithms was ignored. In addition, standard parameters appear to have been used and the influence of the parameter choice on alignment quality was not determined. Finally, as the evaluation was focused on genome alignment, one aspect was not evaluated that becomes relevant for RNA studies in case a mapping algorithm is used that involves transcriptome alignments, such as e.g. RUM or RNASEQR. In this case, the corresponding alignment algorithms have to be able to handle the inherent redundancy resulting from several transcripts of the same gene.

In this study, we address all of these points by performing a comprehensive analysis of 14 algorithms including also read indexing based approaches in the context of RNA-seq experiments. Please note that this is not an evaluation of approaches for assigning reads to their correct position in the transcriptome, but of the underlying alignment algorithms that may be used and, thus, does not focus on splicing detection. For each algorithm, we first determined optimal parameters on smaller training sets for varying read lengths and evaluated stability of the results with parameter changes. On larger test sets, we then evaluated recall and precision as well as runtime and memory requirements. To realistically simulate the conditions observed in RNA-Seq experiments, reads were simulated from transcript sequences. Furthermore, as many of the fastest and best-performing RNA-seq mapping approaches involve transcriptome alignments, simulated reads were aligned to the transcriptome. Our results confirm the superior performance of FM-index based alignment approaches postulated previously and furthermore show that the recently developed representatives of this category have overcome the vulnerability to deviations from the reference sequences due to sequence insertions and deletions.

## Materials and Methods

### Alignment Algorithms

Methods for NGS alignment follow one of two major algorithmic approaches, namely such based on hash tables as known from BLAST and more recent developments based on compressed prefix or suffix array-like structures (FM-index).

#### Hash table based aligners

Algorithms using hash tables build upon quick seeding of alignment candidates which are then extended or discarded using more precise alignment algorithms. In order to quickly find seed locations, either the reference genome (BFAST [11], Novoalign (http://www.novocraft.com), Mosaik (http://code.google.com/p/mosaik-aligner), GNUMAP [12], SHRiMP [13]) or the reads (MAQ [8], RMAP [14], RazerS (www.seqan.de/projects/razers.html)) are split and stored in a hash table. In contrast to BLAST which compares all positions within a window, NGS aligners enhance sensitivity and robustness by using spaced seeds, i.e., multiple windows in which some positions are allowed to deviate from the reference sequence. As a consequence, multiple seed masks are required to cover various permutations of match and mismatch positions. Several strategies exist for the creation of split seed masks. BFAST uses empirically derived optimal seed masks for given read and genome sizes. MAQ generates an exhaustive list of seed masks to allow for retrieval of any sequence with at most 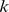 mismatches at the expense of using 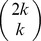 hash tables and aligners such as RMAP and SHRiMP use variations of *q-gram* filtering which gives bounds on the size and number of perfect matches in two strings of given length and total number of edit operations. RMAP takes advantage of this property in order to reduce the number of hash tables and SHRiMP introduces gap-tolerant seeding [13], [14]. Common to all hash table based aligners is the attempt to reduce the search space to a minimum without discarding correct alignment locations. Once seeds are selected, each of the candidate locations is examined by variations of dynamic programming alignment algorithms.

#### FM-index based aligners

Prefix- and suffix tree based algorithms sacrifice error tolerance for extremely fast retrieval of perfect matches. Since all prefixes/suffixes are represented by top-down paths in such a tree, substring matching corresponds to finding a path representing the query, starting at the root [15]. The drawback of using these algorithms is the prohibitively large memory requirement for the uncompressed tree structure, with constants of 15–20 bytes per base of the reference [16]. Subsequent developments took advantage of suffix arrays enhanced by additional information for linear-time substring matching, reducing memory requirements to less than 10 bytes per base [17]. This family of algorithms did not become popular until the development of the FM-index which is a compressed, yet searchable suffix array-like structure [15] from the Burrows-Wheeler transform [18] of the genome. Bowtie [9] is the first and most widely used representative of this class and uses an index of about 2.4 GB for the human genome. BWA [19] and SOAP2 [20] are further popular aligners of this category which greatly outperform implementations using non-compressed structures. The most recent addition is Bowtie 2 [21], which was developed with a particular focus on gapped read alignment. Following seeding of exact substring matches, the algorithms differ substantially in their way of handling mismatches and gaps. The most widely used FM-index based alignment algorithms Bowtie and BWA simply use a distance cutoff for the alignment of the entire read to the genome. Bowtie 2 combines the ultrafast FM-index-based seeding with efficient extension by dynamic programming in order to obtain gapped alignments. Usage specifics of the evaluated mapping algorithms are further described in the Supporting Information (Text S1).

### Selection of Alignment Software for Evaluation

Popular mapping algorithms were selected based on community discussions and the software wiki on http://seqanswers.com. Other criteria for inclusion were free availability, handling of standard formats for input and output, operability in batch mode, active maintenance and documentation that allowed to set up a functional installation with a reasonable effort. Furthermore, an attempt was made to select aligners such that the major algorithmic classes described above were covered. Table 1 lists and categorizes the mapping tools that underwent full evaluation. A comprehensive list of examined software including evaluated parameters and algorithms that could not be evaluated can be found in Text S1.

**Table 1:** Alignment software undergoing complete evaluation.

Algorithmic Class	Aligner	Version
**Hash Table Based Algorithms**		
**Read Indexing**	MAQ	0.7.1
	RazerS	1.1
	RMAP	2.05
**Reference Indexing**	BFAST	0.6.4e
	Genomemapper	0.4.3s
	GNUMAP	2.2.3
	Mosaik	0.7.1
	mrFast	2.0.0.5
	Novoalign	2.07.06
	SHRiMP	2.1.1
**Prefix/Suffix Matching Algorithms**		
**FM-Index Based**	Bowtie	0.12.7
	Bowtie 2	2.0.0-beta7
	BWA	0.5.9-r16
	SOAP2	2.21

Popular alignment tools for whole-genome applications were chosen to represent the major algorithmic classes.

### Simulation of RNA Sequencing Reads

RNA-Seq reads were simulated based on the ENSEMBL human transcriptome (GRCh37) using dwgsim 0.1.2 from the DNAA package (http://dnaa.sf.net). Paired-end reads of 36, 72 and 100 bp with an inner distance (distance between the 3′ ends of the reads) of 250 bp (standard deviation 50 bp) were sampled uniformly from the transcripts using the empirical sequencing error models provided by MetaSim (http://ab.inf.uni-tuebingen.de/software/metasim/). The error model derived from 62 bp Illumina reads was used for the 36 and 72 bp reads, and the error model derived from 80 bp Illumina reads for the 100 bp reads. Default parameters were used otherwise. Novel structural variants were not generated as our study focused on nucleotide-level performance of the evaluated aligners which are not designed to address other tasks than sequence matching. Simulation was based on the transcriptome rather than the genome for a realistic simulation of RNA sequencing experiments and in order to avoid a bias from features that are not generally encountered in RNA-Seq experiments, e.g. repetitive heterochromatin sequences.

### Experimental Setup

**Figure 1:**
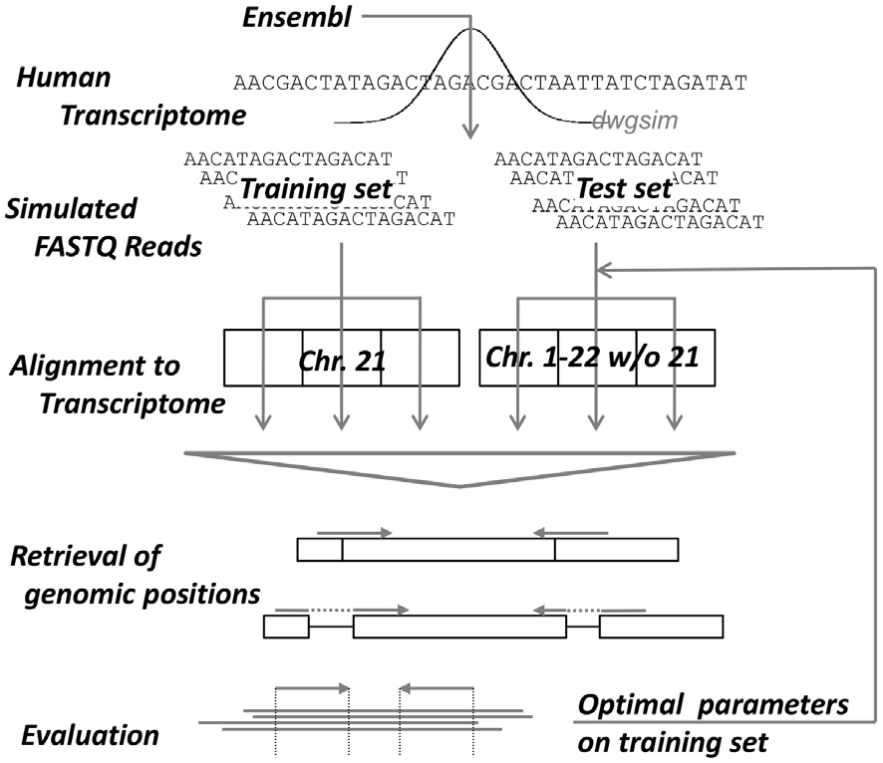
Workflow of alignment evaluation. Reads were simulated from chromosome 21 of the ENSEMBL human transcriptome using dwgsim and aligned to the transcriptome with various parameter combinations for each alignment tool. The best parameter combination for each aligner was selected based on 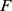-measure first and runtime and memory consumption second in case of ties. The best parameters were then used to evaluate the alignment algorithms on larger test sets simulated from chromosomes 1-22, excluding chromosome 21.

As NGS alignment is a computationally expensive task, exhaustive evaluation of alignment parameters on full-size experimental data sets is infeasible. Therefore, we applied a two-step process in which optimal parameter settings from a pre-defined parameter space (Text S1) were first identified on a smaller training set and then performance was evaluated using these optimal parameter settings on a larger test set. Training and testing was performed independently for each read length evaluated. The training sets contained 500,000 paired end reads each of length 36, 72 and 100 from annotated transcripts on human chromosome 21 (2,212 transcript sequences). The test sets contained 5 million 36, 72 and 100 bp paired end reads, respectively, from transcripts of chromosomes 1-22 excluding chromosome 21 (197,611 transcript sequences). Chromosome 21 was not included in the test set to avoid overlaps in the transcripts on which the simulated reads were based on. Reads were then aligned back to the transcript sequences they were simulated from. Following alignment of the simulated reads to the transcriptome, positions were mapped back to the genome in order to distinguish multiple isoform mappings to the same genomic location from ambiguous mappings to multiple different locations. The full experimental setup is illustrated in Figure 1.

### Performance Measures

#### Precision and recall

Alignment quality was assessed in terms of alignment precision and recall. Precision evaluates which fraction of aligned reads is aligned correctly and recall evaluates which fraction of overall reads were correctly recovered. Recall was ignored in the previous evaluation of alignment algorithms by Ruffalo *et al.* [10]. However, as alignment algorithms may obtain high precision at the cost of very low recall or vice versa, we aimed to evaluate the trade-off between the two performance measures for each algorithm.

To calculate precision and recall, the number of true and false positive alignments was determined. Here, only alignments to distinct genomic positions were evaluated. Alignments mapping the read to the correct genomic location were counted as true positives (TP). Alignments of a read to an incorrect location counted as false positives (FP). Reads that could not be aligned to the correct position counted as false negative alignments (FN). If a read was aligned only to wrong positions, it increased the FN count by one and the FP count by the number of its wrong alignments. Thus, the sum of TP and FN yields the number of reads. Since every read had exactly one correct location of origin and should therefore be mappable, there was no measure for true negative alignments. Note that for this purpose, all alignments provided by the algorithm for the specific parameter choice were evaluated. No additional cut-off was applied except filtering parameters provided by the algorithms themselves. Thus, if an alignment algorithm identified multiple locations for a read, the correct alignment was counted as a true positive and all other alignments to distinct genomic locations as false positives. In general, however, the number of false positive alignments for a particular read was at most 1.

Precision and recall were calculated using the following equations:
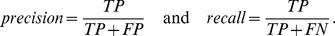



We also calculated the 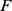-measure, which evaluates the trade-off between precision and recall:
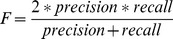



When multiple parameter sets for one aligner resulted in equal 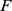-measures in the training step, the best combination was determined as the one having a minimal geometric mean of runtime and memory ranks.

#### Impact of parameter choice on alignment quality

In addition to alignment quality for the best parameter, the large number of parameter combinations tested allowed us to investigate the impact of parameter changes on alignment performance. Dispersion of the 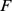-measure for different parameter settings was used as a measure of sensitivity to changes in parameter values. The ratio between average absolute deviation to the median was chosen as measure of dispersion 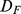:
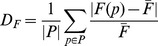
with 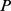 being the parameter space searched for each aligner and 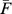 the median of the 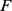-measure over 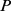. Thus, dispersal was calculated over the F-scores obtained for any evaluated parameter combination.

#### Runtime and memory requirements

Alignment software was installed and run on the BioQuant computer cluster (CentOS 5.4 ×86_64 2.6.18, openMPI 1.2.7rc2 managed by Torque/Maui) with gcc 4.1.2. If multithreading was supported, 8 cores were used. Runtime and memory usage were capped at 10 CPU days and 16 GB, respectively. Total CPU time and memory usage were extracted from PBS job scheduler reports. In particular, for memory usage we extracted the maximum memory used during execution of the job. As the resource usage was only sampled at specific intervals, this value should be considered only as an approximation.

## Results

### Performance on Training Set

Optimal parameter combinations for each of the 14 evaluated aligners and paired-end reads of three different lengths were determined by testing all permutations that appeared to have an impact on alignment quality. The search space was constrained based upon literature research and the respective software documentation. A total of 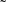
7000 parameter combinations (including alignment quality thresholds provided by the algorithms) were evaluated on the training set of 500,000 reads simulated from chromosome 21. The optimal parameter sets from the training runs along with corresponding performance measures are shown in Table S1. The 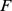-measures for the optimal parameters on the training set ranged between 0.996 (Bowtie2) and 0.7 (RazerS, RMAP). Precision and recall of most aligners were well balanced and memory and runtime requirements varied considerably, without an apparent trade-off between the two or with alignment quality. As neither the genome size nor the number of reads were varied at this stage, there was no apparent difference between the two classes of hash table based aligners.

### Parameter Stability

The evaluation of different parameter permutations also allowed us to assess the robustness of the alignment quality to parameter variations. While a large number of parameters (and combinations thereof) promise tunability of the algorithm to a specific problem, the danger of overwhelming the user with complexity should not be underestimated. Ideally, parameter variation should allow the user to trade precision for recall or to alter runtime and memory properties without affecting overall performance. Table 2 shows the dispersion values of the 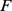-measure over the chosen parameter space for the 72 bp read set. Low dispersion (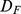) as observed for Bowtie 2 or RazerS indicates that the choice of parameters has only little impact on alignment performance. Algorithms with a high 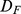 value, such as BFAST, Novoalign and SOAP2 had a particularly wide distribution of alignment quality.

**Table 2:** Parameter Sensitivity.

Aligner	Parameters Tested	D_F_
**Hash Table Based Algorithms**	Read Indexing	
**MAQ**	145	0.05
**RazerS**	162	0.003
**RMAP**	48	0.1
	Reference Indexing	
**BFAST**	6	0.40
**Genomemapper**	216	0.07
**GNUMAP**	324	0.03
**Mosaik**	108	0.01
**mrFast**	7	1.07
**Novoalign**	288	0.22
**SHRiMP**	432	0.06
**Prefix/Suffix Matching Algorithms**	FM-Index Based	
**Bowtie**	180	0.04
**Bowtie 2**	864	0.004
**BWA**	16	0.04
**SOAP2**	72	0.37

Dispersion 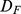 of the 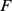-measure across all parameter settings tested for each alignment algorithm is shown for the 72 bp read length training set from human chromosome 21. 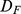 describes the sensitivity of an alignment algorithm to parameter changes. For BFAST and Mosaik, not all available parameters were investigated due to the modular structure of the application.

**Figure 2:**
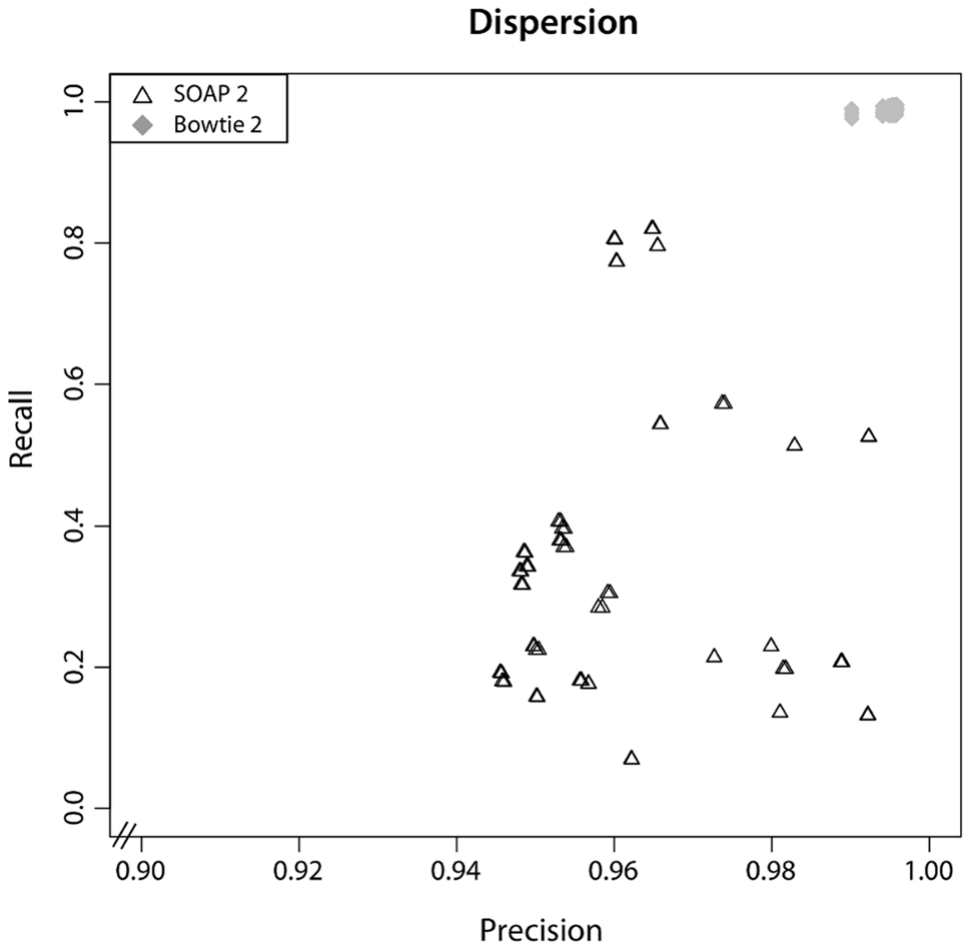
Influence of parameter selection. Precision (x-axis) and recall (y-axis) are shown for SOAP2 (black triangles) and Bowtie 2 (gray diamonds) alignment tools at a read length of 72 bp. Different parameters had only little impact on the alignment performance of Bowtie 2 whereas the recall of SOAP2 was scattered widely. This is reflected by the high 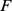-measure dispersion value of SOAP2.

This is exemplified by precision-recall analysis of Bowtie 2 and SOAP2, two aligners with extremely low and high 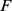-measure dispersion, respectively (Figure 2). Precision remained largely unaffected for both aligners, however SOAP2 had a widely scattered recall distribution. These results also illustrate the importance of evaluating both precision and recall. In terms of precision there is little difference between the algorithms and parameter settings and only the evaluation of recall showed this large variation. Remarkably, the high 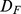 value of mrFast also results from a dramatic drop in recall if the -best flag is not used, which restricts the output to the alignment with the minimum edit distance. This is rather counter-intuitive, as one would expect a large number of false positives but not false negatives if more alignments are outputted. One possible explanation is that in paired-end mode the usage of the -best flag has additional effects such as the utilization of the average paired-end span. Thus, although dispersion makes no statement about the overall performance of the alignment software, it provides an indication whether optimal performance can be achieved without in-depth understanding of algorithmic details.

### Overall Performance

The best-performing parameter combinations on the training set for each alignment algorithm and read length were applied to the test sets of 5 million paired-end reads each simulated from chromosomes 1-22 (excluding chromosome 21). The results of this large-scale test are shown in Table 3. In most cases, if the overall alignment quality in terms of 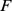-measure was reduced this was due to a lower recall, i.e. a high proportion of reads that could not be aligned to any location. This is observed most strongly for RMAP, which has above-average precision (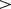
0.91) but very low recall (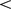
0.53). Remarkably, there was no correlation between F-measure on the one hand and runtime and memory requirements on the other hand. The latter two depended mostly on the algorithmic design principle (read or reference hashing or FM-index), while high F-measures were observed for algorithms of all types.

**Table 3:** Evaluation on test set.

Aligner	Reads	Precision	Recall	F	Memory	Runtime
**Hash Table Based Algorithms**					Read Indexing	
	36	0.913	0.908	0.911	3.1G	2∶20 h^1^
**MAQ**	72	0.922	0.918	0.920	3.4G	2∶20 h^1^
	100	0.925	0.920	0.923	3.7G	2∶30 h^1^
	36	0.883	0.980	0.929	8.8G	77∶05 h
**RazerS**	72	–	–	–	–3	–4
	100	–	–	–	–3	–4
	36	0.912	0.483	0.631	5.4G	18∶00 h
**RMAP**	72	0.919	0.503	0.650	6.0G	10∶30 h
	100	0.920	0.524	0.668	6.0G	7∶05 h
					Reference Indexing	
	36	0.898	0.577	0.703	5.2G	2∶20 h
**BFAST**	72	0.898	0.801	0.847	6.0G	9∶30 h
	100	0.910	0.819	0.862	6.3G	18∶10 h
	36	0.853	0.953	0.900	3.7G	3∶10 h
**Genomemapper** 2	72	0.870	0.967	0.916	3.8G	32∶40 h
	100	0.877	0.971	0.921	3.8G	20∶10 h
	36	–	–	–	–	–4
**GNUMAP** 2 **/Mosaik** 1	72	–	–	–	–	–4
	100	–	–	–	–	–4
	36	–	–	–	–3	–
**mrFast**	72	–	–	–	–3	–
	100	–	–	–	–3	–
	36	0.914	0.914	0.914	2.6G	1∶45 h
**Novoalign**	72	0.921	0.919	0.920	2.6G	3∶20 h
	100	0.924	0.923	0.924	2.6G	4∶20 h
	36	–	–	–	–	–4
**SHRiMP**	72	–	–	–	–	–4
	100	–	–	–	–	–4
**Prefix/Suffix Matching Algorithms**					FM-Index Based	
	36	0.908	0.895	0.902	750M	0∶30 h
**Bowtie**	72	0.919	0.896	0.907	750M	0∶30 h
	100	0.923	0.874	0.898	750M	0∶35 h
	36	0.912	0.911	0.912	1.3G	2∶10 h
**Bowtie 2**	72	0.920	0.919	0.920	1.3G	3∶20 h
	100	0.923	0.922	0.923	1.3G	2∶30 h
	36	0.912	0.875	0.893	1.3G	0∶35 h
**BWA**	72	0.921	0.915	0.918	1.5G	1∶10 h
	100	0.955	0.955	0.955	1.6G	1∶00 h
	36	0.891	0.853	0.872	2.9G	0∶15 h
**SOAP2**	72	0.886	0.751	0.812	2.9G	1∶00 h
	100	0.907	0.834	0.869	2.9G	1∶00 h

Evaluation of optimal alignment parameters on 5 million reads from chromosomes 1-22, excluding 21. Runtime and memory caps were set at 10 CPU days and 16G, respectively. Processes exceeding these limits were killed and partial results were not evaluated. Superscripts indicate the following:

1 Runtime and memory consumption could not be recorded accurately because of the modular structure of the application which makes the automated evaluation of all parameter combinations difficult;

2 Supports only single-end alignment;

3 Exceeded the memory cap of 16G;

4 Exceeded the runtime cap of 10 CPU days.

Unfortunately, RazerS on the 72 or 100 bp test sets as well as MrFast on all tests sets exceeded the 16 GB memory cap and, thus, could not be evaluated. Similarly, GNUMAP, Mosaik and SHRiMP required more than 10 CPU days and could also not be evaluated. As the latter three algorithms used reference indexing, it is not surprising that the size of the reference constituted the problem and not the size of the read set to be aligned. A test set of the same size, but only sampled from chromosome 1 transcripts and aligned only this chromosome, could be aligned within the time and memory limits by all three algorithms (Table S2).

Although runtime and memory requirements varied widely among hash table based aligners, both hashing strategies were clearly outperformed with regard to memory consumption and runtime by the FM-index based approaches. These generally required less than 1.5G memory and 1 h runtime even for the 100 bp set to obtain a comparable precision and recall. Among these, Bowtie in particular was characterized by very low memory consumption and runtime at a reasonable alignment quality, thus supporting the wide-spread use of Bowtie within many RNA-seq mapping approaches. However, Bowtie’s alignment recall was surpassed by both Bowtie 2 and BWA, in particular for longer reads. In contrast to Bowtie, their recall actually improved with read length, likely due to the fact that they were developed with a particular focus on longer sequencing reads. Here, BWA outperformed Bowtie 2 for the longest 100 bp reads, whereas Bowtie 2 had a much higher recall for the short 36 bp reads. This improved alignment accuracy for long reads compared to Bowtie, however, came at the cost of a significantly increased runtime by a factor of 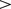
2 and 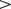
4 for BWA and Bowtie 2, respectively. It should be noted here that although read length is constantly increasing, alignment of very short reads still remains important for RNA-seq as many transcriptome mapping approaches predict novel splice sites by aligning smaller fragments of reads.

### Error Tolerance

In the previous study by Ruffalo *et al.* [10] error tolerance of alignment algorithms was evaluated but only depending on the overall error rate in the simulated read set. However, for any fixed error rate, the set of reads is a mixture of reads with different number of mismatches to the reference. Thus, in this study we aimed to analyze the performance of the algorithms for a specific number of errors or indels in the reads. For this purpose, we determined for each read the number of sequence mismatches or indels, i.e. its error profile. The applied error model and some aligners distinguished between the number of point mutations and the number of sequencing errors by their different distribution throughout the read, however, SNPs were extremely rare compared to sequencing errors, hence their impact on alignment quality was not considered separately.

**Figure 3:**
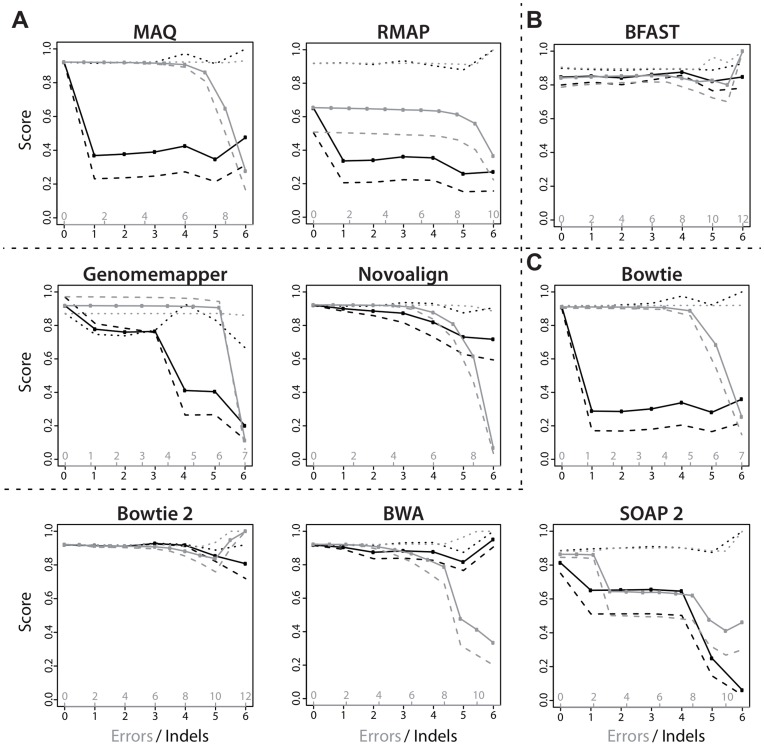
Sensitivity of alignment algorithms to errors and indels. The dependence of alignment precision (dotted lines 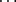), recall (dashed lines 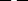) and 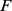-measure (solid lines –) on the number of sequencing errors (gray) and indels (black) was evaluated for each alignment algorithm analyzed in this study. In this figure, results are shown for the 72 bp long reads from the test set (5 million reads), excluding those algorithms which exceeded the memory and runtime cap. Alignment algorithms were sorted by algorithmic design: (A) Read indexing based aligners, (B) Reference indexing based aligners, (C) FM-Index based aligners.

Figure 3 shows the impact of errors on alignment quality for the simulated 72 bp read set from chromosomes 1-22 (excluding 21) for each alignment algorithm using the optimal parameters on the training set, excluding only those algorithms which exceeded the memory or runtime cap. Results for other read lengths are similar. The highest number of mismatches and indels in a correctly aligned read was 12 and 6, respectively. A total of 12 reads (0.0001%) were not aligned by any of the algorithms due to extensive deviation from the reference. Generally, we found that precision of the alignments was only little affected by the number of errors. In contrast, the number of reads that could actually be aligned, i.e. the recall, often dropped drastically with increasing number of errors. Interestingly, distinctive differences could be observed for sequence mismatches on the one hand and indels on the other. Due to the high rate of sequencing errors in NGS applications, alignment algorithms are generally designed to be robust to a small number of single-base mismatches depending on the parameter settings. As a consequence, for most algorithms recall stayed relatively constant in the range of mismatches that were tolerated by the algorithm but then dropped significantly as soon as this range was left.

In contrast to simple mismatches, few algorithms tolerate indels by being designed to handle gapped alignments. As a consequence, recall of most algorithms was impaired considerably by indels. Remarkable tolerance to indels, with near-constant performance as indel counts increased, was shown by BFAST, Bowtie 2 and BWA. While the baseline recall of BFAST was already rather low at 0.81, Bowtie 2 and BWA combined this high indel tolerance with very high recall values. In contrast to BWA, Bowtie 2 not only tolerated indels extremely well but also sequencing errors. Among the ultrafast FM-index based alignment algorithms, Bowtie was most vulnerable to indels. Here, already one indel resulted in a reduction of recall to as little as 0.2. This reduced error and indel tolerance likely explains the reduction in overall recall compared to Bowtie 2 and BWA. It also suggests that it is advisable to replace Bowtie by Bowtie 2 or BWA within a mapping approach in case a substantial number of indels is expected and the additional runtime overhead can be tolerated.

## Discussion

In this study, we performed a comparison of read alignment algorithms with a particular focus on RNA-seq applications. This is not an evaluation of mapping methods, which also identify spliced reads and have been evaluated elsewhere [5], [7], but aims at evaluating the underlying alignment algorithms that are required for any mapping approach. To better reflect the characteristics of RNA-seq experiments, simulation of reads was based on transcript sequences and not genome sequences. As at least for known transcripts alignment of reads to the transcriptome generally outperforms de-novo splicing detection approaches [5], [6], alignment algorithms were evaluated on the task of transcriptome alignment.

A major difficulty in comparing the performance of different aligners is to set up a fair comparison in terms of the task and parameters chosen as well as evaluation metrics. In particular, the choice of the parameters can influence the results dramatically. In the recent study by Ruffalo *et al.* [10], for instance, only default parameters appear to have been evaluated. As the choice of parameters also influences runtime and memory consumption, we aimed to evaluate a more realistic setting in which parameters were tuned to the task at hand. To avoid a bias in parameter selection, optimal parameters were trained on a smaller training set and these optimal parameters were then used for evaluation.

In this way, we found that the largest differences regarding alignment quality were not observed in the precision of the alignments but in recall, in particular with increasing error rates. This fits well with the results of Rufallo *et al.* [10] who observed that alignment accuracy generally increased if low-quality alignments were discarded. In our study, no additional filtering of low-quality alignments were performed but parameter settings of the alignment algorithms were explored in the training step that determine the number and quality of outputted alignments. For Bowtie, for instance, such parameters settings would be the maximum number of mismatches allowed in the seed region and the total number of mismatches allowed in the alignment. Thus, alignment quality was determined only by the alignments which fulfill these constraints for the evaluated algorithm. This highlights the importance of making use of the filtering parameters of alignment algorithms and analyzing recall in addition to precision.

The most striking differences we found in this study concerned the memory and runtime requirements of hash table based aligners compared to the FM-index based algorithms Bowtie, Bowtie 2, BWA and SOAP2. The latter algorithms generally performed as good as the best hash-table based aligners on standard tasks with a resource profile that can be provided by a desktop machine. This shows that the unquestioned use of Bowtie by all state-of-the-art mapping approaches is largely justified as even more recently developed hash-based aligners (e.g. SHRiMP) cannot compete with FM-index based ones in runtime or memory requirements.

Interestingly, analysis of error profiles demonstrated that hash table based aligners can now be replaced by FM-index based aligners even for applications where a large number of mismatches or indels are expected. This includes, for instance, sequencing of species for which only a closely related genome is known or cancer transcriptomics in which differences from the reference are expected and relevant to pathology. Among FM-index based aligners, BWA and, in particular, Bowtie 2 showed a remarkable robustness to insertions and deletions. Thus, although both create an overhead in memory and runtime compared to Bowie, one of them might nevertheless be a better choice in applications in which a substantial number of indels is expected. In this way, they provide a trade-off between the high tolerance to errors and indels of some hash-based aligners and the dramatically reduced runtime and memory requirements of Bowtie.

This analysis also illustrates the importance of evaluating alignment quality compared to the actual number of mismatches and indels in a read and not the average error rate. Our results showed that the reduction of recall due to mismatches and indels is not a gradual process. Instead, as long as the number of differences between read and reference is below a certain amount that can still be tolerated by the algorithm only little changes are observed. However, as soon as this amount is exceeded, recall generally drops dramatically. Furthermore, as only 11% of reads in our analysis had 4 or more base mismatches and only 0.2% of reads contained indels, error- and in particular indel-tolerance had only a small effect on average alignment quality.

### Conclusions

In summary, this study is relevant for scientists involved in the analysis of RNA-Seq data in several respects. First, it provides a comprehensive evaluation of the performance of state-of-the-art algorithms from all major algorithmic classes used for read alignment. Second, it provides guidance with regard to the choice of algorithm and parameters. Although the task at hand may differ from the situations simulated in this study, the relative performance of the algorithms with regard to alignment quality, runtime and memory consumption as well as their weaknesses and advantages can be extrapolated from this study to other tasks. Third, this study establishes a procedure for identifying optimal parameters using a smaller training set and highlights the importance of evaluating both recall and precision and considering the actual number of mismatches and indels in a read instead of overall error rates. Finally, even if the the alignments algorithms are not used as stand-alone procedures but as integral parts of sophisticated mapping approaches for identifying spliced reads, assessment of their performance is of major importance. As alignment algorithms are generally used in a generic fashion, exchanging the underlying alignment procedure for a better performing one provides one straightforward way to improve the overall performance of a mapping strategy. Here, our study supports to some degree the de-facto standard of using Bowtie as alignment algorithm within RNA-seq mapping pipelines, but also illustrates the potentials of replacing it by other FM-index based algorithms such as BWA and, in particular, Bowtie 2.

## Supporting Information

Table S1Table of optimal parameter sets for each alignment algorithm as determined on a test set of 500,000 reads simulated from transcripts of chromosome 21. The best parameter combination for each aligner was selected based on 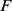-measure (see main text), run time and memory usage in case of ties.(PDF)

Table S2 Evaluation results for alignment algorithms based on reference hashing on a test set simulated from and aligned to chromosome 1 (5 million reads).
(PDF)

Text S1 Details on the algorithms that were examined for this study.
(PDF)
