# Supplementary material for: A Comprehensive Evaluation of Alignment Algorithms in the Context of RNA-Seq
Source: PLoS One. 2012 Dec 26;7(12):e52403. doi: 10.1371/journal.pone.0052403 (PMC3530550; doi:10.1371/journal.pone.0052403)
Supplement: Table S1 — Table of optimal parameter sets for each alignment algorithm as determined on a test set of 500,000 reads simulated from transcripts of chromosome 21. The best parameter combination for each aligner was selected based on -measure (see main text), run time and memory usage in case of ties. (PDF) [file pone.0052403.s001.pdf]

Table S1. Optimal parameter sets for each alignment algorithm as determined on a test set of 500,000 reads simulated from transcripts of chromosome 21. The best parameter combination for each aligner was selected based on F-measure (see main text), run time and memory usage in case of ties. Please refer to Text S1 for a detailed explanation of the parameters.

Aligner	Reads	Parameter String	F	Memory	Runtime	Parameters Tested
**Hash Table Based Algorithms**						Read Indexing
**MAQ**	36	m=0.005, n=3, e=120, C=5 -W	0.992	n/a[1]	n/a[1]	145
	72	m=0.01, n=3, e=120, C=5	0.995	n/a[1]	n/a[1]	145
	100	m=0.005, n3, e=120, C=5	0.993	n/a[1]	n/a[1]	145
**RazerS**	36	m=15, i=86, dr=0, rr=99	0.985	730M	8.05 min	162
	72	m=1, i86, dr=0, rr=99	0.927	1.1G	23.13 min	162
	100	m=15, i=94, dr=0, rr=95, -id	0.701	1.7G	32 min	162
**RMAP**	36	m=6, M=15, S=4, h=7, -Q	0.708	700M	14.3 min	48
	72	m=6, M=15, S=4, h=7, -Q	0.722	789M	19.5 min	48
	100	m=6, M=15, S=4, h=7, -Q	0.731	722M	22.2 min	48
						Reference Indexing
**BFAST**	36	a=4	0.859	1.2G[1]	10 min[1]	6
	72	a=4	0.859	1.3G[1]	38 min[1]	6
	100	a=4	0.892	1.7G[1]	55 min[1]	6
**Genomemapper**[2]	36	s=8, M=4, G=1, n=5, -h, -w	0.984	1.1G	1.8 min	216
	72	s=10, M=6, G=3, n=5	0.988	1.1G	2.2 min	216
	100	s=10, M=6, G=1, n=5, -w	0.989	1.1G	1.3 min	216
**GNUMAP**[2]	36	m=10, a=0.2, M=0, s=0, j=5, bin=6	0.978	394M	9.6 min	324
	72	m=14, a=0.2, M=0, s=0, j=9, bin=6	0.984	453M	24.4 min	324
	120	m=14, a=0.2, M=0, s=0, j=9, bin=8	0.986	400M	22.1 min	324
**Mosaik**[1]	36	hs=10, act=4, mm=3, bw=21	0.872	87M	5.5 min	108
	72	hs=10, act=4, mm=5	0.940	140M	27.8 min	108
	120	hs=13, act=0, mm=5	0.962	460M	50 min	108
**mrFast**	36	ws=12, e=6, –best	0.923	2.1G	140 min	7
	72	ws=12, e=6, –best	0.914	2.4G	145 min	7
	100	ws=12, e=6, –best	0.909	2.4G	136 min	7
**Novoalign**	36	t150, Q0, R6, -rAll	0.995	228M	14 min	288
	72	t150, Q0, R2, -rAll	0.984	220M	19 min	288
	100	t=150, Q=0, R=6, -rAll	0.987	1G	21 min	288
**SHRiMP**	36	sw=14, w=160, n=4, h=68, a=10	0.908	1G	10.5 min	432
	72	sw=14, w=160, n=4, h=76, a=8	0.903	1GM	29.1 min	432
	100	sw=16, w=160, n=4, h=76, a=8, –strata	0.927	1G	40 min	432
**Prefix / Suffix Matching Algorithms**						FM-Index Based
**Bowtie**	36	n=3, e=90	0.988	6M	1.2 min	180
	72	n=3, e=90	0.986	26M	1.3 min	180
	100	n=3, e=90	0.972	31M	1.5 min	180
**Bowtie 2**	36	rdg=7,3, mp=4,1, L=18, N=1	0.993	145M	7 min	864
	72	rdg=7,5, mp=8,1, L=20, N=1	0.996	155M	14 min	864
	100	rdg=7,3, mp=8,4, L=22, N=1	0.993	165M	18 min	864
**BWA**	36	n=8, o=2, e=3	0.977	5M	1.1 min	16
	72	n=8, o=4, e=5	0.992	230M	2.3 min	16
	100	n=8, o=4, e=5	0.993	300M	2 min	16
**SOAP2**	36	M=4, r=1, v=7, g=2	0.963	683M	1 min	72
	72	M=4, r=1, v=7, g=4	0.887	693M	6.2 min	72
	100	M=4, r=1, v=7, g=4	0.941	785M	6.6 min	72

[1] Runtime and memory usage could not be recorded accurately because of the modular structure of the application which makes the automated evaluation of all parameter combinations difficult.
[2] Supports only single-end alignment.
