# Supplementary material for: A Comprehensive Evaluation of Alignment Algorithms in the Context of RNA-Seq
Source: PLoS One. 2012 Dec 26;7(12):e52403. doi: 10.1371/journal.pone.0052403 (PMC3530550; doi:10.1371/journal.pone.0052403)
Supplement: Table S2 — Evaluation results for alignment algorithms based on reference hashing on a test set simulated from and aligned to chromosome 1 (5 million reads). (PDF) [file pone.0052403.s002.pdf]

Table S2. Evaluation results for alignment algorithms based on reference hashing on a test set simulated from and aligned to chromosome 1 (5 million reads). Runtime and memory caps were set at 10 CPU days and 16G, respectively. Processes exceeding these limits were killed and output – if produced – was evaluated. Superscripts indicate the following: [1] supports only single-end alignment; [2] exceeded the memory cap of 16G.

Aligner	Reads	Precision	Recall	F	Memory	Runtime
					Reference Indexing	
**BFAST**	36	0.932	0.804	0.864	1.3G	0:45 h
	72	0.933	0.832	0.880	1.6G	4:00 h
	100	0.926	0.817	0.868	1.9G	17:50 h
**Genomemapper**[1]	36	0.926	0.978	0.951	1.3G	50:20 h
	72	0.833	0.866	0.849	1.3G	31:20 h
	100	0.972	0.953	0.992	1.3G	13:50 h
**GNUMAP**[1]	36	0.926	0.984	0.954	1.5G	28:15 h
	72	0.943	0.984	0.965	1.4G	38:45 h
	100	0.939	0.920	0.959	1.4G	39:20 h
**Mosaik**	36	0.881	0.792	0.834	234M	3:00 h
	72	0.884	0.951	0.917	392M	21:50 h
	100	0.912	0.876	0.950	740M	15:00 h
**mrFast**	36	–	–	–	–[2]	–
	72	–	–	–	–[2]	–
	100	–	–	–	–[2]	–
**Novoalign**	36	0.977	0.977	0.977	1.2G	1:25 h
	72	0.965	0.964	0.965	1.2G	3:20 h
	100	0.955	0.955	0.955	1.2G	4:20 h
**SHRiMP**	36	0.846	0.926	0.884	1.7G	40:10 h
	72	0.838	0.773	0.805	2G	115:30 h
	100	0.830	0.928	0.876	2.6G	156:10 h
