# Supplementary material for: A Comprehensive Evaluation of Alignment Algorithms in the Context of RNA-Seq
Source: PLoS One. 2012 Dec 26;7(12):e52403. doi: 10.1371/journal.pone.0052403 (PMC3530550; doi:10.1371/journal.pone.0052403)
Supplement: Text S1 — Details on the algorithms that were examined for this study. (PDF) [file pone.0052403.s003.pdf]

1 ALIGNMENT SOFTWARE NOTES

Details on the algorithms that were examined for this study. If they were included in the evaluation, the most important parameters and those used for optimization are explained. If they could not be evaluated, the reasons for not including them are provided. Please refer to the respective manuals for more details.

1.1 BFAST

Version 0.6.4e, used for final evaluation.
Only the postprocessing step was optimized
Available from http://bfast.sf.net.
FastQ (after minor reformatting for paired end reads) and SAM compatible

BFAST contains an option to build in the absence of bzip headers and libraries, however many users report I/O errors in this setting. This issue is still present at the time of writing (version 0.7.0).

Workflow

bfast fasta2brg: conversion of reference genome from fasta to BRG.
bfast index: create index of the reference genome
parameters:

Parameter	Values	Description	Values
-w	[INT]	hash width	14
-d	[INT]	Split the index into 4^d to save memory	default
-m	[STRING]	Seed masks (newline-separated strings of 1 and 0)	default*

∗: Following seed masks are suggested for reads over 40 bp by the author of BFAST:

```
1111111111111111111111
1111101110111010100101011011111
1011110101101001011000011010001111111
10111001101001100100111101010001011111
11111011011101111011111111
111111100101001000101111101110111
11110101110010100010101101010111111
111101101011011001100000101101001011101
1111011010001000110101100101100110100111
1111010010110110101110010110111011
```

bfast match: seeding of candidate alignment locations (CAL)
parameters:

Parameter	Values	Description	Values
-K		Ignore multiple CAL producing indices	not used
-M		Ignore multiple CAL producing reads	not used

bfast localalign: Local alignment of CAL
parameters:

Parameter	Values	Description	Values
-u		ungapped alignment	not used
-U		extend matches independently from seed constraints	not used

bfast postprocess: Filtering of alignments
parameters:

Parameter	Values	Description	Values
-a	[0-4]	filtering mode	0,1,3,4
-m	[0-255]	mapping quality cutoff for a=1	15,30,60

Filtering mode 4 had to be used because it was the only one reporting multiply aligning reads.

1.2 Bowtie

Version 0.12.7, used for final evaluation.
Many parameters involved in performance tuning were not evaluated and are not presented here.
Available from http://bowtie-bio.sf.net.
Fully FastQ and SAM compatible.

Workflow

bowtie index: Build compressed FM-index of the reference.
bowtie: align reads to reference
parameters:

Parameter	Values	Description	Values
-v	[INT]	SOAP mode - allow up to v mismatches total	4,6,8
-n	[INT]	MAQ mode - allow up to n in the first l bases	2,3
-l	[INT]	in MAQ mode: number of bases to consider	default
-e	[INT]	allow mismatches with total FASTQ score of -e	50, 70, 90
-k	[INT]	maximum number of alignments to report	15, 5
-a		report all alignments, mutually exclusive with k	
–best		order alignments by score	
-m		report only if unique, mutually exclusive with k	
–strata		report all best scoring alignments	

Various combinations of output filtering flags were tested.

1.3 Bowtie 2

Version 2.0.0-beta7, used for final evaluation.
Many parameters involved in performance tuning were not evaluated and are not presented here
Available from http://bowtie-bio.sourceforge.net/bowtie2/.
Fully FastQ and SAM compatible

Workflow

bowtie2 index: Build compressed FM-index of the reference.
bowtie2: align reads to reference
parameters:

Parameter	Values	Description	Values
-N	[0,1]	Allowed mismatches per seed	0, 1
-L	[INT]	Seed substring length. Decrease for sensitivity	18, 20, 22
–mp	[INT,INT]	Range of mismatch penalties	$[4,6,8]\times[1,2,4]$
–rdg	[INT,INT]	Read gap open and extension penalty	$[3,5,7]\times[1,2,3,5]$
–rfg	[INT,INT]	Reference gap open and extension penalty	same as rdg
–local	[FLAG]	Enable soft clipping within alignment	

1.4 BWA

Version 0.5.9-r16, used for final evaluation.
Available from http://bio-bwa.sf.net.
Fully FastQ and SAM compatible

Workflow

bwa index: Build compressed FM-index of the reference.
bwa aln: align reads to reference
parameters:

Parameter	Values	Description	Values
-n	[INT—FLOAT]	maximum edit distance from reference (absolute if INT, relative if FLOAT)	2,4,6,8
-o	[INT]	maximum # gaps	2,4
-e	[INT]	maximum gap extensions	3,5
-d	[INT]	disallow deletions in d bases at $3'$ end	not used
-l	[INT]	use only l bases from the $5'$ end	not used

1.5 Genomemapper

Version 0.4.3s, used for final evaluation.
Available from http://1001genomes.org/.
Fully FastQ compatible, supports only single-end alignment. BED output format.

Workflow

gmindex: index the reference

Parameter	Values	Description	Values
-s	[INT]	seed length	8, 10, 12

genomemapper: align reads to reference
parameters:

Parameter	Values	Description	Values
-G	[INT]	maximum # gaps	1,3
-M	[INT]	maximum # mismatches	2, 4, 6
-a		report all alignments	
-n	[INT]	report up to n randomly selected alignments	5, 15
-h		perform extension beyond the seed match region	
-w		de-filter: report best alignment regardless of M and G	

1.6 GNUMAP

Version 2.2.3, used for final evaluation.
Available from http://dna.cs.byu.edu/gnumap/.
Fully FastQ compatible, supports only single-end alignment. SAM output lacks header.

Workflow

gnumap: index reference and align reads (reference can be saved on disk)
parameters:

Parameter	Values	Description	Values
-m	[INT]	seed size	8, 10, 14
-a	[FLOAT]	minimum alignment score (relative to maximum if -p set)	0.05, 0.1, 0.2
-M	[INT]	maximum # gaps	0, 2, 4
-s	[INT]	space between indexing windows	0, 1
-j	[INT]	skip $\frac{m}{2} + j$ bases when aligning the reads	-2, 0, 2
–binsize	[INT]	resolution of the alignment	6, 8

1.7 GMAP/GSNAP

Version 2010-07-27, not used because much processing was required and output is not comparable.
Available from http://research-pub.gene.com/gmap/.
Requires much pre-processing for index and read construction in the correct format. Claims to be SAM compatible.
Algorithm does not accept individual contigs as reference, rather performs gapped alignment of each read and thereby claims to discover exon-spanning reads. Splice site and SNP databases can be provided and manual appeared promising. However, it was impossible to make the software run in a reasonable amount of time.

1.8 MAQ

Version 0.7.1, used for final evaluation.
Available fr.om http://maq.sf.net/.
Fully FastQ compatible, supports only single-end alignment. Perl script provided to convert from MAQ format to SAM which lacks header. Read names are truncated and need to be recovered for alignment evaluation.

Workflow

maq fastq2bfq: convert sequences to binary format
maq match: align reads
parameters:

Parameter	Values	Description	Values
-m	[FLOAT]	expected rate of difference between reads and reference	0.001, 0.005, 0.01
-n	[INT]	allow n mismatches in first 24 nt of read	1, 2, 3
-C	[INT]	# of mapping locations to report for each read	5, 15
-e	[INT]	maximum sum of mismatch base qualities	30, 60, 90, 120
-W		Disable full Smith-Waterman alignment	

1.9 Mosaik

Version 0.7.1, used for final evaluation.
Available from http://code.google.com/p/mosaik-aligner/.
Fully FASTQ compatible. Provides a converter for SAM format.

Workflow

MosaikBuild: Generate binary representations of reads and reference
MosaikJump: Create jump database for compressed representation of the reference
parameters:

Parameter	Values	Description	Values
-hs	[INT]	seed size	10, 13, 15
-mhp	[INT]	Maximum number of locations saved per hash value	100

MosaikAlign: Align reads to reference
parameters:

Parameter	Values	Description	Values
-hs	[INT]	seed size	10, 13, 15
-mhp	[INT]	Maximum number of locations saved per hash value	100
-act	[INT]	alignment candidate threshold	-4, 0, 4
-mm	[INT]	maximum # mismatches	0, 3, 5, 7
-bw	[INT]	bandwidth for banded Smith-Waterman	17, 21

1.10 mrFast

Version 2.0.0.5, used for final evaluation.
Available from http://mrfast.sf.net.
Fully FastQ compatible. SAM output lacks header and contains illegal characters in CIGAR strings.

Workflow

mrfast –index: Build index of the reference.
parameters:

Parameter	Values	Description	Values
-ws	[INT]	window size, recommended: $\left\lfloor \frac{len}{err+1} \right\rfloor$	12

mrfast –search: align reads to reference
parameters:

Parameter	Values	Description	Values
-e	[INT]	maximum edit distance	2,4,6
–best		report only best scoring alignments	

Only one window size could be tested at a time because mrFast will always use the same file name to store the reference index.

1.11 Novoalign

Version 2.07.06, used for final evaluation.
Available from http://novocraft.com/.
Fully FastQ and SAM compatible.

Workflow

novoindex: Index the reference
parameters:

Parameter	Values	Description	Values
-k	[INT]	seed size	14
-s	[INT]	space between indexing windows	1

novoalign: Align reads to the reference
parameters:

Parameter	Values	Description	Values
-t	[INT]	minimum alignment quality	50, 100, 150
-Q	[INT]	minimum quality for reporting	0, 20, 60
-R	[INT]	Minimum score difference between best alignments to treat read as non-repetitive	0, 2, 4, 6
-r	[STRING]	mulitple alignment treatment mode	All, Exhaustive {1,5,15}, 0.85, 0.95

Novoalign has a vast number of parameters and only the ones chosen for variation are shown.

1.12 RazerS

Version 1.1, used for final evaluation.
Available from http://www.seqan.de/projects/razers.html.
Fully FastQ compatible. Own BED-like output format which had to be parsed separately.

Workflow

razers: align reads to reference

parameters:

Parameter	Values	Description	Values
-m	[INT]	output at most m of the best matches	1, 5, 15
-i	[INT]	required % identity for reporting	86, 90, 94, 98
-dr	[INT]	consider alignments that have dr more errors than the best	0,1,2
-rr	[INT]	recognition rate	95,99,100
–id		allow indels	

1.13 RMAP

Version 2.05, used for final evaluation.
Available from http://rulai.cshl.edu/rmap/.
Extensive reformatting of FastQ input necessary for paired-end reads. 6 column BED output that requires special attention with strand information.

Workflow

rmap: align reads to reference
parameters:

Parameter	Values	Description	Values
-m	[INT]	maximum # of mismatches	0,2,4,6
-M	[INT]	report at most M best-scoring multiple mappings	5,15
-S	[INT]	# of layered seeds	3,4
-A		align pairs simultaneously to use their location information	always used
-Q		Quality matching	

Wildcard matching mode -W, supposedly an improvement over -Q mode, did not run properly.

1.14 Segemehl

Version Jan 14 13:11:37 CET 2010, not used for final evaluation.
Available from http://www.bioinf.uni-leipzig.de/Software/segemehl/.
Fully FastQ compatible, supports only single-end alignment. Non-standard output format.
Usage appears straightforward but this suffix array-based algorithm did not manage to hold a 1.2 megabase transcriptome (619 sequences) in memory due to the large data structure. Therefore, training and evaluation could not be carried out on chromosomes 21 and 1 (3.6 and 30 megabases), respectively.

1.15 SHRiMP

Version 2.1.1, used in final evaluation.
Available from http://compbio.cs.toronto.edu/shrimp/.
Fully FastQ and SAM compatible

Workflow

gmapper-ls -S: Build and save index of the reference.
parameters:

Parameter	Values	Description	Values
-s	[STRING]	load pre-defined seed masks with given weights	w12, w14, w16 -H
-H		Hash k-mers into 24 bit strings to limit size to 4^{12}	used with -s w16

gmapper-ls -L: load reference and align reads to it
parameters:

Parameter	Values	Description	Values
-w	[INT]	window size (in % of read length)	120, 140, 160
-n	[2,3,4]	Seed matching mode for paired end	2,3,4
-U		ungapped	not used
-h	[INT]	SW-score to keep alignment	60, 68, 76
-a	[INT]	width of banded Smith-Waterman search	8, 10
–strata		report only the best scoring alignments	
–half-paired		report reads with only one end mapping	

SHRiMP provides a manual to split the genome index into smaller chunks which was not necessary in this study.

1.16 SOAP

Version 2.21, used for final evaluation.
Available from http://soap.genomics.org.cn/.
Fully FastQ compatible. Comes with a Perl script to convert SOAP output to SAM.

Workflow

2bwt-builder: Build compressed FM-index of the reference.
soap: align reads to reference
parameters:

Parameter	Values	Description	Values
-l	[INT]	consider only l bases from $5'$ end	not used
-s	[INT]	minimum alignment length	not used
-v	[INT]	allow up to v mismatches	3,5,7
-g	[INT]	allow one gap of size g	0,2,4
-M	[0,1,2,4]	seed match mode [0,1,2 mismatches, 4: best hit]	0,1,2,4
-r	[0,1,2]	reporting of multiple mappings	1,2
